# Annual Address

**Published:** 1897-03

**Authors:** David W. Cheever

**Affiliations:** Boston


### ANNUAL ADDRESS.[1]

BY DAVID W. CHEEVER, M.D., BOSTON.

Mr. President and Gentlemen,—It is rather to my shame that it can be said that this is my second visit to you, although you have done me the honor to give me membership for some years. It is, however, great pleasure for me to say a few words to-night, and possibly I might allude to a time—not very far distant—when I was still a teacher in the Harvard Medical School, and it was a part of my province to give some lectures on the surgery of the mouth, and to those lectures the members of the Dental School were requested to come. Some of you, I dare say, may remember that time, and some of you are, perhaps, of too recent date to have ever heard me say anything about it.

It was my earnest endeavor at that time to teach about the mouth those things which it seemed to me that every one ought to know who practised your profession, in order to guard against all sorts of ills both to yourselves and to the patients who fall into your hands. I endeavored to be particularly careful to discriminate between those things which were important in that part of the body and those things which were not important, and to show that it was of vital importance that the dentist should be able to recognize the early stages of certain diseased conditions of which he might warn his patients knowingly and understandingly in order that they might either correct their lives to prevent those affections or seek the advice of physicians and surgeons in order to have them treated.

I must say as I listened to Dr. Donald and his eloquent description of the charlatan in former years, who went about in a chariot and worked such destruction in the mouths of innocent people in the places he visited, that I felt myself a little twinge of remorse for what I had done in my earlier practice. In those days I extracted a great many teeth, and I am ashamed to say a great many teeth that ought to have been preserved. When I was younger and was one of the junior physicians of the Boston Dispensary, it was the duty of the physicians there to extract the teeth of every one who complained of toothache, and who said he wanted it done, without much discrimination as to whether it was really necessary.

---

[1] Delivered at the Annual Meeting of the American Academy of Dental Science, Boston, November, 1896.

I make this confession in order to touch upon a point that I wish to make clear,—the very great change that has taken place through your means in the treatment of the teeth of the poor. The poor people who cannot go and have their teeth properly looked after and repaired at the time it is most needed were formerly entirely neglected, and it is due to your efforts and to your charity that clinics, so to speak, have been built up among the poor; that dental dispensaries have been established, where something may be done for the teeth of poor people besides having them pulled out the moment they ache.

Moreover,—and this is of greater importance perhaps, because it refers not only to the poor, but to many who are well-to-do,—your profession has done an incalculable amount of good by instructing people in the importance of proper care of the teeth. It is a familiar fact to physicians that many immigrants who come to our shores, some in the first and some in the second generation, begin to lose their teeth. The healthy immigrant from Ireland no sooner enters upon domestic service than she begins to lose her color, the character of the teeth become impaired, there is a gradual decline in health and strength, and they become a ready prey to all forms of disease. As a natural consequence, the next generation of those people are generally poorly provided with teeth and health and strength, and, this being the fact, anything that will tend to correct the conditions leading up to these results is not only so much gain to them, but probably also to their descendants in future years; and it seems to me that no more useful work is done to-day in Boston than that which you do in endeavoring to care for the teeth of the poor people, thereby supplementing and many times obviating the work of the physicians and surgeons at our hospitals. Through the charity of professional and philanthropic people, our hospital service is now so well arranged that the poor laborer who gets knocked over in the street and brought in with a fractured or dislocated limb receives, free of charge, more thorough care and treatment than he would in his own home; and perhaps as high medical and surgical skill as can be obtained anywhere by the millionaire, and anywhere in the United States. And it is in the same line with this class of work that your infirmary service belongs, which enables the infirmary patient also to enjoy privileges fully as important to the health and comfort of the individual.

I will allude again, but only for a moment, to the fact which your orator called your attention to,—the interest which your profession must have felt in the late celebration of the fiftieth anniversary of the first public use of an anæsthetic for the prevention of pain; and I shall not attempt to emphasize how prominently it must have appeared that the practical demonstration of the fact that anæsthesia could be so used that severe surgical operations could be borne without pain, was made by one of your profession; and for this we certainly owe you a debt of gratitude.

Now with regard to the future. It seems to me that two great problems present themselves to you in the matter of the care and preservation of the teeth of the entire community,—two special fields towards which you ought to devote your energies, your industry, your most careful thought and investigation. The first of these is, that you instruct your patients with regard to the proper selection of articles of food and diet which will tend to preserve the teeth. Many of us think that since the wheat was bolted into flour there has been a loss of bone-forming qualities, a loss of phosphates, which is entailed by the process of bolting. We think we find abundant proof of this in the change which can be noticed in the teeth and the general health of the Irish and Scotch immigrants after they have lived a few years in this country. Their food in the old countries consisted mainly of oatmeal and the cereals and articles of food made from coarse grains, which they promptly neglect when they get here, seeking always the finest wheaten flour, which they do not always properly masticate, but succeed in swallowing by the aid of large quantities of tea. To the fine white flour and to the teapot, I believe, must be ascribed the various disorders of the system, the conditions of chronic indigestion and dyspepsia, into which a great many of these young women pass.

The other point that I had in mind is a matter which is yet in the infancy of its progress, and that is the study of bacteriology in order that we may contend with those very numerous microbes or parasites which are the concomitants or probably the causes of the decay of the teeth. It seems to me that with the microscope and with the various germicides which are coming into use there is a great field open to you, the earnest study of which will have an important bearing on the preservation of teeth from decay.

To sum up these points briefly, then, they are: first, that people must be instructed with regard to the kind of food which is best not only for their teeth, but for their general health as well; and, second, that in the study of bacteria, you will learn how to oppose and prevent their growth and stop the consequent destruction of the teeth.

